# Association of Serum 25-Hydroxyvitamin D Concentration with Pulmonary Function in Young Adults

**DOI:** 10.3390/nu10111728

**Published:** 2018-11-11

**Authors:** Vanda Craveiro, Maria Cabral, Joana Araújo, Helena Falcão, João Tiago Guimarães, Elisabete Ramos

**Affiliations:** 1 EPIUnit—Instituto de Saúde Pública, Universidade do Porto, Rua das Taipas, nº 135, 4050-600 Porto, Portugal; joana.araujo@ispup.up.pt (J.A.); jtguimar@med.up.pt (J.T.G.); eliramos@med.up.pt (E.R.); 2 Unidade de Epidemiologia—Departamento de Ciências da Saúde Pública e Forenses e Educação Médica da Faculdade de Medicina da Universidade do Porto, 4200-319 Porto, Portugal; maria.cabral@ispup.up.pt; 3 Serviço de Imunoalergologia do Centro Hospitalar do Porto, E.P.E., 4099-001 Porto, Portugal; falcao.helena@gmail.com; 4 Serviço de Patologia Clínica do Centro Hospitalar de São João, E.P.E., 4200-319 Porto, Portugal; 5 Departamento de Biomedicina da Faculdade de Medicina da Universidade do Porto, 4200-319 Porto, Portugal

**Keywords:** vitamin D, 25-hydroxyvitamin D, pulmonary function, spirometry, young adults

## Abstract

The role of vitamin D on pulmonary function is unclear and is mostly studied in patients, smokers and elderly people. The aim of this paper was to evaluate the association between serum 25-hydroxyvitamin D [25(OH)D] concentration and pulmonary function in young adults. Cross-sectional analysis of 499 individuals that were evaluated at 21 years of age as part of the population-based cohort Epidemiological Health Investigation of Teenagers in Porto (EPITeen). Serum 25(OH)D was categorized according to the Institute of Medicine. Pulmonary function was evaluated using spirometry. Linear regression models were used to estimate the regression coefficients (β) and its 95% confidence intervals (95% CI), and were adjusted for confounders. Education, smoking, body mass index, and season of evaluation were determinants of serum 25(OH)D concentration. Prevalence of serum 25(OH)D concentration <50 nmol/L was 48.9%. A decrease in all pulmonary function parameters, with the decrease of serum 25(OH)D, was observed. The higher effect was found for peak expiratory flow (PEF). Having as reference participants with serum 25(OH)D concentration ≥50 nmol/L, PEF was significantly lower for those with a concentration of 30 to <50 nmol/L (β= −0.576; 95% CI: −0.943, −0.210), and for those with a concentration of <30 nmol/L (β= −0.650; 95% CI: −1.155, −0.146). Although only PEF attained statistical significance, the consistent results with the other parameters support the role of serum 25(OH)D to promote better pulmonary function in young adults.

## 1. Introduction

Calcitriol is the metabolite responsible for the biological actions of vitamin D, described as genomic, mediated via the vitamin D-receptor (VDR) transcriptional effects in the nuclei of target cells, and non-genomic, mediated via the rapid VDR-induced signal transduction pathways on the cell membrane and/or cytoplasm [1,2,3,4].

The ubiquitous presence of VDR in human tissues contributes to support the role of calcitriol across several tissues in the organism [1,2]. These actions include the suppression of cell growth, regulation of apoptosis, induction of cell differentiation, and modulation of immune responses [2]. The pathways in which vitamin D acts on the pulmonary function could be related with its anti-inflammatory effects in the airway, both modulating innate and adaptive immunity, and with its capacity to regulate airway remodeling by the modulation of fibroblasts, inhibition of matrix metalloproteinases, and also inhibiting growth of airway smooth muscle cells [5,6].

The relationship of vitamin D with pulmonary function has been investigated and no consistent results were found. There are studies which observed a positive association between serum 25(OH)D concentration and pulmonary function parameters in individuals from the general population, namely children [7], adolescents [8], adults [9,10,11,12], and elderly men [13]. However, another population-based study suggested that serum 25(OH)D is not an important determinant of pulmonary function in adults [14]. Additionally, some data supports that the role of vitamin D could be related with other conditions that influence the pulmonary function, such as tobacco [15,16] or allergic diseases [17,18,19].

The present study intends to fill the existent gap in the literature on the relationship of vitamin D and pulmonary function in early adulthood, when the peak of pulmonary maturation is attained [20]. Thus, it was aimed to evaluate the association of serum 25(OH)D concentration with pulmonary function in young adults.

## 2. Materials and Methods 

This study was developed as part of the population-based cohort Epidemiological Health Investigation of Teenagers in Porto (EPITeen). As reported elsewhere [21], the EPITeen included adolescents born in 1990, who were enrolled at public and private schools in Porto, Portugal, during the 2003/2004 school year. Two subsequent evaluations were performed by contacting directly the participants or their legal guardians. The second evaluation took place when participants were on average 17 years old (2007/2008) and the third evaluation occurred when participants were on average 21 years old (2011–2013). 

In all three evaluations, the procedures were similar and were performed by a team of trained health professionals, comprising questionnaires (demographic, social, behavioral and clinical characteristics of the participants and their families), physical evaluation (anthropometrical measurements and spirometry), and the collection of a blood sample after overnight fasting.

The study complies with the Declaration of Helsinki and was approved by the National Commission of Data Protection and by the Ethics Committee of the Hospital de São João (31 July 2012). Procedures to guarantee data confidentiality and protection were assured. Participants received written and oral information explaining the purpose and the design of the study, and signed the written informed consent.

### 2.1. Participants

At the recruitment, 2786 eligible participants were identified and 2159 agreed to participate, resulting in an overall participation rate of 77.5%. From the 2159 participants at baseline, 1716 (79.5%) were re-evaluated at 17 years and 1318 (61.0%) were re-evaluated at 21 years. From the 1204 subjects that performed the three evaluations, due to budgetary constraints, serum 25(OH)D concentration was determined in a sub-sample of 559 individuals (these 559 subjects were randomly chosen among the ones that have a blood sample collected in all three evaluations) [22]. From those, 58 participants without assessment of pulmonary function at 21 years and two pregnant women were excluded. Since none participants reported having a disease or a treatment with a major impact on pulmonary function and/or vitamin D status, our final sample included 499 participants. Participants included in the analysis were compared to those not included. Both groups were similar, but non-included participants presented significantly lower education and were more sedentary during leisure time (Table 1).

### 2.2. Serum 25(OH)D Evaluation

Serum total 25(OH)D concentration was determined using a direct competitive chemiluminescence immunoassay (DiaSorin LIAISON^®^, Saluggia, Italy). This assay uses magnetic particles (solid phase) coated with antibody against 25(OH)D and 25(OH)D conjugated to an isoluminol derivative (tracer). During the first incubation phase (10 min), 25(OH)D is dissociated from binding protein by buffer containing 10% ethanol and then binds to the anti-25(OH)D antibody on the solid phase. After a second 10 min incubation with the tracer, the unbound material is washed off and starter reagents are added to generate a flash chemiluminescent signal which is measured by a photomultiplier and is inversely related to 25(OH)D concentration [23]. The criteria proposed by the Institute of Medicine [1] was used to categorize the serum 25(OH)D concentration as: risk of deficiency (<30 nmol/L), risk of inadequacy (30 to <50 nmol/L), and sufficiency (≥50 nmol/L) of vitamin D.

### 2.3. Pulmonary Function

Pulmonary function was evaluated using a spirometer (SpiroLab^®^, Medical International Research, Rome, Italy) and followed the standards of the American Thoracic Society/European Respiratory Society [24]. The best of three technically accepted forced expirations was considered, and the analysis included forced vital capacity (FVC), forced expiratory volume in the first second (FEV_1_), FEV_1_/FVC ratio, peak expiratory flow (PEF), and mean forced expiratory flow during the middle half of FVC (FEF_25-75_)_._ The technician that performed the spirometries was not aware of the vitamin D status of the participants, since the serum was collected at the same time and analysed after the performance of all evaluations.

### 2.4. Covariates

Data on covariates was collected using self-reported questionnaires. Participants’ education was assessed as the last completed schooling year. Personal history of allergic disease comprised information on rhinitis, asthma and allergy. Participants were asked separately for each disease if it had ever been diagnosed. The possible answers were “yes”, “no” and “do not know”. If at least one of the three diseases had ever been diagnosed, allergic disease was considered (based on personal declaration). Vitamin supplementation use at 21 years of age was assessed asking participants the question “In the last 12 months did you take some vitamins or minerals’ supplements?”. If “yes” the commercial name and dosage of supplementation were registered. Leisure-time physical activity was classified as “sitting”, “standing and/or walking (without running)”, and “very active” [25]. Participants were classified as smokers at least once a day, occasional smokers (smokers less than once a day, former smokers, and just tried) and non-smokers, in relation to their habit at 21 years of age. 

Weight and height were obtained with the subject in light indoor clothes and no shoes. Weight was measured in kilograms, to the decimal, using a digital scale (Tanita TBF-300, Tanita Corporation of America, Inc., Arlington Heights, IL, USA), and height was measured in centimeters, to the decimal, using a stadiometer. Body mass index (BMI) was calculated as weight (kilograms) divided by squared height (meters) and classified according to the World Health Organization [26]. 

Season during which participants were evaluated was combined into two categories: March–October and November–February. It was considered that vitamin D winter period ranges between November and February, since at latitudes above 40° N (latitude of Porto, where the study was conducted) sunlight appears not to be strong enough to the endogenous synthesis, from November to February. This is supported by other works performed at similar latitudes of Porto, namely in Boston (USA) [27] and in Valencia (Spain) [28]. During the other months of the year, the ultraviolet index in Porto is >3, being sufficient for the cutaneous production of vitamin D [29]. Thus, sunny season was considered as March-October.

### 2.5. Statistical Analysis

Continuous variables were tested for Normality by checking both skewness and kurtosis and the histogram. They were presented as mean (standard-deviation) and compared using independent-samples *t* test and one-way ANOVA. The association was tested between serum 25(OH)D and pulmonary function parameters using serum 25(OH)D concentration either as a continuous or as a categorical variable. For both approaches, linear regression models were used to estimate the regression coefficients (β) and their respective 95% confidence intervals (95% CI). Final models were adjusted for those variables with statistical significance on the serum 25(OH)D concentration that remained significant (education, BMI and season), or that it was expected a theoretical effect (leisure-time physical activity), or with theoretical relevance for pulmonary function (sex and height). Since in the analysis with serum 25(OH)D as a categorical variable, the higher category (vitamin D sufficiency) was used as the reference class, in the analysis with serum 25(OH)D as a continuous variable, the values were inverted, so the results from both variables could be directly compared. Statistical significance was considered with an alpha critical value of 0.05. Statistical analysis was performed using IBM^®^ SPSS^®^ Statistics version 24.0 (IBM Corp., Armonk, NY, USA).

## 3. Results

In our sample, mean (standard-deviation) serum 25(OH)D concentration was 54.97 (27.76) nmol/L. Participants’ description of pulmonary function parameters and serum 25(OH)D are presented in Table 2.

The prevalence of the risk of vitamin D deficiency was 14.2% and the risk of vitamin D inadequacy was 34.7%. Table 3 shows serum 25(OH)D according to participants’ characteristics. Mean serum 25(OH)D concentration was lower in less educated participants, in daily smokers, in those who were on the extreme categories (lower and higher) of BMI and in the ones evaluated between November and February.

Table 4 depicts the association of serum 25(OH)D concentration with pulmonary function parameters. After adjustment, a decrease in all pulmonary function parameters was found with the decrease of serum 25(OH)D concentration. However, only for PEF the effect was statistically significant, with a mean 10 mL/s decrease for each nanomole per litre decrease in serum 25(OH)D concentration.

Table 5 displays the association with pulmonary function parameters considering serum 25(OH)D categories. After adjustment, and being considered as reference participants with vitamin D sufficiency, all pulmonary function parameters decreased with the decrease of serum 25(OH)D categories. However, the effect was statistically significant only for PEF, with a mean difference of 576 mL/s between the participants at risk of vitamin D inadequacy and those with vitamin D sufficiency, and with a mean difference of 650 mL/s between the participants at risk of vitamin D deficiency and those with vitamin D sufficiency.

In order to test the role of vitamin D among those with higher probability to have lower pulmonary function, the association was analysed using serum 25(OH)D as a continuous variable (Table 6) and as a categorical variable (Table 7). Statistically significant results were observed among participants with asthma regarding FVC, FEV_1_/FVC ratio, and PEF.

## 4. Discussion

In this study, a prevalence of 34.7% for the risk of vitamin D inadequacy and a prevalence of 14.2% for the risk of vitamin D deficiency were observed. These results are in accordance with the worldwide observation of low vitamin D status [30,31], particularly in Europe [32,33,34], which has also been found in young adults [35,36]. 

In the present dataset of young adults, a decrease in all parameters of spirometry with a decrease in serum 25(OH)D concentration was found. For PEF, a dose-response relationship with serum 25(OH)D concentration was observed, which reflects a possible action of vitamin D in pulmonary function. These results support that vitamin D plays a role in the pulmonary function, even in healthy people, beyond its effects in a disease scenario [10,12,17,18,19,37].

The role of vitamin D is reinforced with the observation that in the group with a higher potential of limited pulmonary function (the asthmatics), the association between serum 25(OH)D concentration and pulmonary function parameters is stronger. These results are in accordance with previous studies in which similar findings were observed [17,18].

In the present study, PEF was the parameter for which the association with serum 25(OH)D achieved statistical significance. By definition, PEF is the “highest flow achieved from a maximum forced expiratory maneuver started without hesitation from a position of maximal lung inflation” [24]. The maximum airflow after a forced expiration could be affected by airway obstruction, which could decrease PEF without changes in FEV_1_ [38]. Vitamin D could promote an increasing PEF by inhibiting the growth of smooth muscle cells in the airway and by modulating the proliferation of fibroblasts, also contributing to the balanced state of matrix metalloproteinases, and in this way, promoting the airflow [5,6]. Moreover, the potential role of vitamin D in modulating inflammation could be one of the explanations for this association [5,6]. 

In this study, the association between PEF and serum 25(OH)D was the most strong and PEF was the only parameter that reached statistical significance. The difficulty to find statistically significant results is explained by the characteristics of our sample—young and healthy, in which the effect of 25(OH)D is expected to be slight. PEF is most affected in those with rhinitis, asthma or allergies, which frequently coexist in this age group [39]. On the other hand, FVC depends on the thoracic corpulence of the individual, which is at its’ maximum at early adulthood [20,40], so only very strong changes would be detected with a significant effect.

This study was based on a biomarker that represents both intake and cutaneous synthesis of vitamin D [29]. During winter, when there is no sufficient ultraviolet index to the cutaneous synthesis of vitamin D [29], the consumption of dietary sources of vitamin D, namely fish, contribute to the maintenance of the vitamin D status [41,42]. In countries with a traditionally higher fish intake, like Portugal, the consumption of fish and/or fish oil may be an important aid to increase the levels of vitamin D, despite not being sufficient to optimize the vitamin D status [41]. The results presented support that there is a need to conduct more studies, to understand the role of each source of vitamin D (diet and sun exposure).

Additional important results were the determinants of serum vitamin D status. As previously reported, more educated participants presented higher mean serum 25(OH)D concentration [43], as well as the subjects evaluated between March and October, reflecting the seasonal variation described for serum 25(OH)D [28]. Participants on the extreme categories (lower and higher) of BMI, had lower mean serum 25(OH)D concentration despite the fact that previous studies found an inverse association [36,44]. In fact, the results for underweight participants could reflect a lower intake of vitamin D along with the low energy intake from the diet. Daily smokers presented lower mean serum 25(OH)D concentration, as shown by Tønnesen R et al. [36], probably reflecting the clustering of unhealthy behaviors such as less intake of food sources of vitamin D and/or lower participation in activities that promote sun exposure. This result is particularly relevant since in our sample, among daily smokers, 63.4% smoke less than 10 cigars per day, 33.6% smoke 11–20 cigars per day, and 3.05% smoke 21–30 cigars per day, thus representing mostly light smokers.

Some limitations should be acknowledged when interpreting the results. Our mean serum 25(OH)D concentration was in the borderline sufficiency status [1], and it is possible that potential beneficial effects of vitamin D might be more expressive at higher levels, which may have contributed to a lower strength of association. Some studies considered the Endocrine Society reference values for vitamin D [45], which are higher than the range values of serum, 25(OH)D, concentration that were used in this research [1]. Besides, the analytical method used to determine serum 25(OH)D concentration varies among studies, with the results being influenced by the type of assay and manufacturer, and there is no consensual method [23]. The laboratory that performed the measurements of serum 25(OH)D did not participate in the international Vitamin D External Quality Assessment Scheme (DEQAS), however, it participated in the Randox International Quality Assessment Scheme (RIQAS), an external quality control program which supports the quality of the measurements performed.

Regarding the study strengths, serum 25(OH)D concentration was used to determine vitamin D status, since it is a biomarker of both intake and cutaneous synthesis of vitamin D [29] and DiaSorin LIAISON^®^ is an accurate and precise method for serum total 25(OH)D determination [23]. Also, objective measures of pulmonary function were assessed. The observed associations were independent of possible confounders—sex, height, education, leisure-time physical activity, BMI and season. The population-based sample used in the present study may enhance generalizability. To our knowledge, this is the first study that evaluates the association between serum 25(OH)D concentration and pulmonary function, targeting early adulthood, when the peak of pulmonary maturation is attained [20]. Since data is cross-sectional, the causative nature of associations cannot be determined; yet, it is not expected that participants have altered their behavior in consequence of the characteristics evaluated. Therefore, one can assume that the association verified did not result because of reverse causality.

## 5. Conclusions

Although only PEF attained statistical significance, the consistent results with the other parameters support the role of serum 25(OH)D concentration to promote better pulmonary function in young adults.

## Figures and Tables

**Table 1:** Description and comparison of the characteristics of non-included and included participants in the present analysis.

	Non-Included (*n* 1265)		Included (*n* 499)		
Characteristics	*n*	%	*n*	%	*p*
Sex					0.586
Female	646	51.1	262	52.5	
Participants’ number of schooling years					0.036
≤12	461	36.9	156	31.3	
13–14	351	28.1	138	27.7	
≥15	439	35.1	205	41.1	
Missing	14		0		
Allergic disease					0.340
With rhinitis or asthma or allergies	476	37.6	200	40.1	
Vitamin supplementation					0.937
Yes	361	28.6	141	28.4	
Missing	1		2		
Leisure-time physical activity					0.047
Sitting	434	34.6	151	30.4	
Standing and/or walking (without running)	540	43.0	246	49.5	
Very active	282	22.5	100	20.1	
Missing	9		2		
Frequency of smoking					0.673
Smoke at least once a day	354	28.4	131	26.3	
Occasional smoker	541	43.4	224	45.0	
Non-smoker	352	28.2	143	28.7	
Missing	18		1		
BMI (kg/m^2^)					0.778
<18.5	76	6.1	29	5.8	
18.5–24.9	871	69.8	339	67.9	
25.0–29.9	232	18.6	99	19.8	
≥30.0	68	5.5	32	6.4	
Missing	18		0		
Season					0.797
March-October	880	69.6	344	68.9	
November-February	385	30.4	155	31.1	

BMI: body mass index.

**Table 2:** Description of participants’ characteristics.

Characteristics	* Mean (SD)
Height, cm	168.4 (8.7)
BMI, kg/m^2^	23.2 (3.8)
Pulmonary function	
FVC (L)	4.45 (0.98)
FEV_1_ (L)	3.96 (0.81)
FEV_1_/FVC ratio (%)	89.5 (6.04)
PEF (L/s)	7.27 (2.29)
FEF_25-75_ (L/s)	4.68 (1.09)
Vitamin D status	
Serum 25(OH)D, nmol/L	54.97 (27.76)
Categories of serum 25(OH)D concentration	***n* (%)**
<30 nmol/L	71 (14.2)
30 to <50 nmol/L	173 (34.7)
≥50 nmol/L	255 (51.1)

25(OH)D: 25-hydroxyvitamin D; BMI: body mass index; FEF_25-75_: mean forced expiratory flow during the middle half of FVC; FEV_1_: forced expiratory volume in the first second; FVC: forced vital capacity; PEF: peak expiratory flow; SD: standard-deviation. * All variables presented a distribution similar to the Normal. Categories of vitamin D status: <30 nmol/L corresponding to risk of deficiency, 30 to <50 nmol/L corresponding to risk of inadequacy, and ≥50 nmol/L corresponding to sufficiency of vitamin D.

**Table 3:** Serum 25(OH)D concentration (nmol/L) according to participants’ characteristics.

	*n*	* Mean (SD)	*p*
Sex			0.495
Male	237	54.08 (29.90)	
Female	262	55.78 (25.70)	
Participants’ number of schooling years			0.039
≤12	156	50.29 (26.89)	
13–14	138	56.90 (29.88)	
≥15	205	57.24 (26.60)	
Allergic disease			0.366
With rhinitis or asthma or allergies	200	53.74 (25.92)	
Without rhinitis or asthma or allergies	299	55.89 (28.95)	
Vitamin supplementation			0.374
Yes	141	56.67 (30.08)	
No	356	54.21 (26.80)	
Leisure-time physical activity			0.115
Sitting	151	50.75 (25.17)	
Standing and/or walking (without running)	246	56.53 (28.97)	
Very active	100	57.83 (28.05)	
Frequency of smoking			0.031
Smoke at least once a day	131	51.03 (27.71)	
Occasional smoker	224	58.49 (28.46)	
Non-smoker	143	53.06 (26.27)	
BMI (kg/m^2^)			0.001
<18.5	29	44.57 (16.24)	
18.5–24.9	339	57.88 (29.79)	
25.0–29.9	99	52.40 (23.90)	
≥30.0	32	41.56 (16.01)	
Season			0.001
March-October	344	57.76 (29.26)	
November-February	155	48.79 (22.99)	

BMI: body mass index; SD: standard-deviation. * All variables presented a distribution similar to the Normal. There are two missing data for vitamin supplementation and leisure-time physical activity and one missing data for frequency of smoking.

**Table 4:** Association of serum 25(OH)D concentration (nmol/L) with pulmonary function (PF) parameters.

	Crude	Adjusted *
PF Parameters	β (95% CI)	β (95% CI)
FVC (L)	0.000 (−0.003, 0.004)	−0.001 (−0.003, 0.000)
FEV_1_ (L)	0.000 (−0.002, 0.003)	−0.001 (−0.003, 0.000)
FEV_1_/FVC ratio (%)	−0.011 (−0.030, 0.008)	−0.004 (−0.022, 0.015)
PEF (L/s)	**−0.008 (−0.016, −0.001)**	**−0.010 (−0.016, −0.004)**
FEF_25-75_ (L/s)	0.000 (−0.004, 0.003)	−0.001 (−0.004, 0.002)

25(OH)D: 25-hydroxyvitamin D; CI: confidence interval; FEF_25-75_: mean forced expiratory flow during the middle half of FVC; FEV_1_: forced expiratory volume in the first second; FVC: forced vital capacity; PEF: peak expiratory flow; PF: pulmonary function. Serum 25(OH)D concentration was analysed as a continuous variable. * Adjusted for sex, height, education, leisure-time physical activity, BMI and season. Statistically significant estimates are in bold.

**Table 5:** Association of serum 25(OH)D categories with pulmonary function (PF) parameters.

			Crude	Adjusted *
PF Parameters	25(OH)D (nmol/L)	*n*	β (95% CI)	β (95% CI)
FVC (L)	<30 nmol/L	71	0.221 (−0.037, 0.478)	−0.034 (−0.168, 0.101)
	30 to <50 nmol/L	173	0.093 (−0.096, 0.281)	−0.061 (−0.159, 0.036)
	≥50 nmol/L	255	Reference	Reference
FEV_1_ (L)	<30 nmol/L	71	0.178 (−0.034, 0.391)	−0.009 (−0.131, 0.113)
	30 to <50 nmol/L	173	0.041 (−0.115, 0.197)	−0.067 (−0.156, 0.022)
	≥50 nmol/L	255	Reference	Reference
FEV_1_/FVC ratio (%)	<30 nmol/L	71	−1.211 (−2.792, 0.371)	−0.383 (−1.908, 1.142)
	30 to <50 nmol/L	173	−1.038 (−2.199, 0.123)	−0.422 (−1.529, 0.685)
	≥50 nmol/L	255	Reference	Reference
PEF (L/s)	<30 nmol/L	71	−0.343 (−0.942, 0.256)	**−0.650 (−1.155, −0.146)**
	30 to <50 nmol/L	173	−0.295 (−0.735, 0.144)	**−0.576 (−0.943, −0.210)**
	≥50 nmol/L	255	Reference	Reference
FEF_25-75_ (L/s)	<30 nmol/L	71	0.043 (−0.243, 0.329)	−0.096 (−0.347, 0.155)
	30 to <50 nmol/L	173	−0.050 (−0.260, 0.160)	−0.141 (−0.323, 0.042)
	≥50 nmol/L	255	Reference	Reference

25(OH)D: 25-hydroxyvitamin D; CI: confidence interval; FEF_25-75_: mean forced expiratory flow during the middle half of FVC; FEV_1_: forced expiratory volume in the first second; FVC: forced vital capacity; PEF: peak expiratory flow; PF: pulmonary function. * Adjusted for sex, height, education, leisure-time physical activity, BMI and season. Statistically significant estimates are in bold. Categories of vitamin D status: <30 nmol/L corresponding to risk of deficiency, 30 to <50 nmol/L corresponding to risk of inadequacy, and ≥50 nmol/L corresponding to sufficiency of vitamin D.

**Table 6:** Association of serum 25(OH)D concentration (nmol/L) with pulmonary function (PF) parameters in participants diagnosed with asthma (*n* 69) and in participants diagnosed with allergy (*n* 164).

	Participants Diagnosed with Asthma	Participants Diagnosed with Allergy
PF Parameters	Crude β (95% CI)	Crude β (95% CI)
FVC (L)	**0.009 (0.001, 0.017)**	0.002 (−0.004, 0.008)
FEV_1_ (L)	0.003 (−0.003, 0.010)	0.001 (−0.004, 0.006)
FEV_1_/FVC ratio (%)	**−0.094 (−0.159, −0.030)**	−0.017 (−0.053, 0.020)
PEF (L/s)	**−0.022 (−0.041, −0.003)**	−0.011 (−0.024, 0.002)
FEF_25-75_ (L/s)	−0.008 (−0.019, 0.002)	0.000 (−0.006, 0.007)

25(OH)D: 25-hydroxyvitamin D; CI: confidence interval; FEF_25-75_: mean forced expiratory flow during the middle half of FVC; FEV_1_: forced expiratory volume in the first second; FVC: forced vital capacity; PEF: peak expiratory flow; PF: pulmonary function. Serum 25(OH)D concentration was analysed as a continuous variable. Statistically significant estimates are in bold.

**Table 7:** Association of serum 25(OH)D categories with pulmonary function (PF) parameters in participants diagnosed with asthma and in participants diagnosed with allergy.

		Participants Diagnosed with Asthma		Participants Diagnosed with Allergy	
PF Parameters	25(OH)D (nmol/L)	*n*	Crude β (95% CI)	*n*	Crude β (95% CI)
FVC (L)	<30 nmol/L	11	**0.808 (0.256, 1.361)**	26	0.239 (−0.201, 0.679)
	30 to <50 nmol/L	25	0.183 (−0.237, 0.604)	57	0.177 (−0.161, 0.515)
	≥50 nmol/L	33	Reference	81	Reference
FEV_1_ (L)	<30 nmol/L	11	0.411 (−0.078, 0.855)	26	0.175 (−0.197, 0.547)
	30 to <50 nmol/L	25	0.049 (−0.290, 0.388)	57	0.103 (−0.183, 0.388)
	≥50 nmol/L	33	Reference	81	Reference
FEV_1_/FVC ratio (%)	<30 nmol/L	11	**−5.512 (−9.988, −1.036)**	26	−1.453 (−4.200, 1.293)
	30 to <50 nmol/L	25	−2.636 (−6.045, 0.772)	57	−1.535 (−3.641, 0.572)
	≥50 nmol/L	33	Reference	81	Reference
PEF (L/s)	<30 nmol/L	11	**−1.412 (−2.696, −0.127)**	26	**−1.222 (−2.200, −0.244)**
	30 to <50 nmol/L	25	−0.624 (−1.602, 0.355)	57	−0.461 (−1.211, 0.289)
	≥50 nmol/L	33	Reference	81	Reference
FEF_25-75_ (L/s)	<30 nmol/L	11	−0.197 (−0.889, 0.495)	26	−0.061 (−0.550, 0.428)
	30 to <50 nmol/L	25	−0.298 (−0.825, 0.229)	57	−0.052 (−0.428, 0.323)
	≥50 nmol/L	33	Reference	81	Reference

25(OH)D: 25-hydroxyvitamin D; CI: confidence interval; FEF_25-75_: mean forced expiratory flow during the middle half of FVC; FEV_1_: forced expiratory volume in the first second; FVC: forced vital capacity; PEF: peak expiratory flow; PF: pulmonary function. Statistically significant estimates are in bold. Categories of vitamin D status: <30 nmol/L corresponding to risk of deficiency, 30 to <50 nmol/L corresponding to risk of inadequacy, and ≥50 nmol/L corresponding to sufficiency of vitamin D.

## References

[1] Institute of Medicine (2011). Dietary Reference Intakes for Calcium and Vitamin D.

[2] Dusso A.S., Brown A.J., Slatopolsky E. (2005). Vitamin D. Am. J. Physiol. Renal Physiol..

[3] Haussler M.R., Jurutka P.W., Mizwicki M., Norman A.W. (2011). Vitamin D receptor (VDR)-mediated actions of 1α,25(OH)_2_ vitamin D_3_: Genomic and non-genomic mechanisms. Best Pract. Res. Clin. Endocrinol. Metab..

[4] Trochoutsou A.I., Kloukina V., Samitas K., Xanthou G. (2015). Vitamin-D in the immune system: Genomic and non-genomic actions. Mini Rev. Med. Chem..

[5] Yawn J., Lawrence L.A., Carroll W.W., Mulligan J.K. (2015). Vitamin D for the treatment of respiratory diseases: Is it the end or just the beginning?. J. Steroid Biochem. Mol. Biol..

[6] Kerley C.P., Elnazir B., Faul J., Cormican L. (2015). Vitamin D as an adjunctive therapy in asthma. Part 1: A review of potential mechanisms. Pulm. Pharmacol. Ther..

[7] Yao T.C., Tu Y.L., Chang S.W., Tsai H.J., Gu P.W., Ning H.C., Hua M.C., Liao S.L., Tsai M.H., Chiu C.Y. (2014). Serum 25-hydroxyvitamin D levels in relation to lung function and exhaled nitric oxide in children. J. Pediatr..

[8] Tolppanen A.M., Williams D., Henderson J., Lawlor D.A. (2011). Serum 25-hydroxy-vitamin D and ionised calcium in relation to lung function and allergen skin tests. Eur. J. Clin. Nutr..

[9] Black P.N., Scragg R. (2005). Relationship between serum 25-hydroxyvitamin D and pulmonary function in the third national health and nutrition examination survey. Chest.

[10] Berry D.J., Hesketh K., Power C., Hypponen E. (2011). Vitamin D status has a linear association with seasonal infections and lung function in British adults. Br. J. Nutr..

[11] Choi C.J., Seo M., Choi W.S., Kim K.S., Youn S.A., Lindsey T., Choi Y.J., Kim C.M. (2013). Relationship between serum 25-hydroxyvitamin D and lung function among Korean adults in Korea National Health and Nutrition Examination Survey (KNHANES), 2008–2010. J. Clin. Endocrinol. Metab..

[12] Afzal S., Lange P., Bojesen S.E., Freiberg J.J., Nordestgaard B.G. (2014). Plasma 25-hydroxyvitamin D, lung function and risk of chronic obstructive pulmonary disease. Thorax.

[13] van Schoor N.M., de Jongh R.T., Daniels J.M., Heymans M.W., Deeg D.J., Lips P. (2012). Peak expiratory flow rate shows a gender-specific association with vitamin D deficiency. J. Clin. Endocrinol. Metab..

[14] Shaheen S.O., Jameson K.A., Robinson S.M., Boucher B.J., Syddall H.E., Sayer A.A., Cooper C., Holloway J.W., Dennison E.M. (2011). Relationship of vitamin D status to adult lung function and COPD. Thorax.

[15] Lange N.E., Sparrow D., Vokonas P., Litonjua A.A. (2012). Vitamin D deficiency, smoking, and lung function in the Normative Aging Study. Am. J. Respir. Crit. Care Med..

[16] Larose T.L., Brumpton B.M., Langhammer A., Camargo C.A., Chen Y., Romundstad P., Mai X.M. (2015). Serum 25-hydroxyvitamin D level, smoking and lung function in adults: The HUNT Study. Eur. Respir. J..

[17] Sutherland E.R., Goleva E., Jackson L.P., Stevens A.D., Leung D.Y. (2010). Vitamin D levels, lung function, and steroid response in adult asthma. Am. J. Respir. Crit. Care Med..

[18] Gupta A., Sjoukes A., Richards D., Banya W., Hawrylowicz C., Bush A., Saglani S. (2011). Relationship between serum vitamin D, disease severity, and airway remodeling in children with asthma. Am. J. Respir. Crit. Care Med..

[19] Niruban S.J., Alagiakrishnan K., Beach J., Senthilselvan A. (2015). Association between vitamin D and respiratory outcomes in Canadian adolescents and adults. J. Asthma.

[20] Mindell J., Chaudhury M., Aresu M., Jarvis D., Craig R., Mindell J. (2011). Lung function in adults. Health Survey for England 2010.

[21] Ramos E., Barros H. (2007). Family and school determinants of overweight in 13-year-old Portuguese adolescents. Acta Paediatr..

[22] Cabral M., Araujo J., Teixeira J., Barros H., Martins S., Guimaraes J.T., Lopes C., Ramos E. (2016). Vitamin D levels and cardiometabolic risk factors in Portuguese adolescents. Int. J. Cardiol..

[23] Wagner D., Hanwell H.E., Vieth R. (2009). An evaluation of automated methods for measurement of serum 25-hydroxyvitamin D. Clin. Biochem..

[24] Miller M.R., Hankinson J., Brusasco V., Burgos F., Casaburi R., Coates A., Crapo R., Enright P., van der Grinten C.P., Gustafsson P. (2005). Standardisation of spirometry. Eur. Respir. J..

[25] Magalhães A., Severo M., Autran R., Araújo J., Santos P., Pina M.F., Ramos E. (2017). Validation of a single question for the evaluation of physical activity in adolescents. Int. J. Sport Nutr. Exerc. Metab..

[26] (2000). WHO Obesity: Preventing and Managing the Global Epidemic.

[27] Webb A.R., Kline L., Holick M.F. (1988). Influence of season and latitude on the cutaneous synthesis of vitamin D_3_: Exposure to winter sunlight in Boston and Edmonton will not promote vitamin D_3_ synthesis in human skin. J. Clin. Endocrinol. Metab..

[28] Serrano M.A., Canada J., Moreno J.C., Gurrea G. (2017). Solar ultraviolet doses and vitamin D in a northern mid-latitude. Sci. Total Environ..

[29] EFSA NDA Panel (2016). Scientific opinion on dietary reference values for vitamin D. EFSA J..

[30] Holick M.F. (2010). The vitamin D deficiency pandemic: A forgotten hormone important for health. Public Health Rev..

[31] Hilger J., Friedel A., Herr R., Rausch T., Roos F., Wahl D.A., Pierroz D.D., Weber P., Hoffmann K. (2014). A systematic review of vitamin D status in populations worldwide. Br. J. Nutr..

[32] Spiro A., Buttriss J.L. (2014). Vitamin D: An overview of vitamin D status and intake in Europe. Nutr. Bull..

[33] Cashman K.D., Dowling K.G., Skrabakova Z., Gonzalez-Gross M., Valtuena J., De Henauw S., Moreno L., Damsgaard C.T., Michaelsen K.F., Molgaard C. (2016). Vitamin D deficiency in Europe: Pandemic?. Am. J. Clin. Nutr..

[34] Manios Y., Moschonis G., Lambrinou C.P., Tsoutsoulopoulou K., Binou P., Karachaliou A., Breidenassel C., Gonzalez-Gross M., Kiely M., Cashman K.D. (2018). A systematic review of vitamin D status in southern European countries. Eur. J. Nutr..

[35] Tangpricha V., Pearce E.N., Chen T.C., Holick M.F. (2002). Vitamin D insufficiency among free-living healthy young adults. Am. J. Med..

[36] Tonnesen R., Hovind P.H., Jensen L.T., Schwarz P. (2016). Determinants of vitamin D status in young adults: Influence of lifestyle, sociodemographic and anthropometric factors. BMC Public Health.

[37] Pfeffer P.E., Hawrylowicz C.M. (2018). Vitamin D in Asthma: Mechanisms of Action and Considerations for Clinical Trials. Chest.

[38] Akar H.H., Tahan F., Gungor H.E. (2015). The association of forced expiratory volume in one second and forced expiratory flow at 50% of the vital capacity, peak expiratory flow parameters, and blood eosinophil counts in exercise-induced bronchospasm in children with mild asthma. Asia Pac. Allergy.

[39] Gough H., Grabenhenrich L., Reich A., Eckers N., Nitsche O., Schramm D., Beschorner J., Hoffmann U., Schuster A., Bauer C.P. (2015). Allergic multimorbidity of asthma, rhinitis and eczema over 20 years in the German birth cohort MAS. Pediatr Allergy Immunol..

[40] Lum S., Stocks J. (2010). Forced expiratory manoeuvres. Eur. Respir. Monogr..

[41] Lehmann U., Gjessing H.R., Hirche F., Mueller-Belecke A., Gudbrandsen O.A., Ueland P.M., Mellgren G., Lauritzen L., Lindqvist H., Hansen A.L. (2015). Efficacy of fish intake on vitamin D status: A meta-analysis of randomized controlled trials. Am. J. Clin. Nutr..

[42] Handeland K., Skotheim S., Baste V., Graff I.E., Froyland L., Lie O., Kjellevold M., Markhus M.W., Stormark K.M., Oyen J. (2018). The effects of fatty fish intake on adolescents’ nutritional status and associations with attention performance: Results from the FINS-TEENS randomized controlled trial. Nutr. J..

[43] Jaaskelainen T., Knekt P., Marniemi J., Sares-Jaske L., Mannisto S., Heliovaara M., Jarvinen R. (2013). Vitamin D status is associated with sociodemographic factors, lifestyle and metabolic health. Eur. J. Nutr..

[44] Vimaleswaran K.S., Berry D.J., Lu C., Tikkanen E., Pilz S., Hiraki L.T., Cooper J.D., Dastani Z., Li R., Houston D.K. (2013). Causal relationship between obesity and vitamin D status: Bi-directional Mendelian randomization analysis of multiple cohorts. PLoS Med..

[45] Holick M.F., Binkley N.C., Bischoff-Ferrari H.A., Gordon C.M., Hanley D.A., Heaney R.P., Murad M.H., Weaver C.M., Endocrine S. (2011). Evaluation, treatment, and prevention of vitamin D deficiency: An Endocrine Society clinical practice guideline. J. Clin. Endocrinol. Metab..

